# Epidemiology and Clinical Outcomes of Cardiac Arrhythmias in Pulmonary Arterial Hypertension

**DOI:** 10.1016/j.chpulm.2024.100132

**Published:** 2024-12-25

**Authors:** Thanaboon Yinadsawaphan, Mustafa Suppah, Srekar N. Ravi, Juan M. Farina, Robert L. Scott, Dan Sorajja

**Affiliations:** a Department of Cardiovascular Diseases, Mayo Clinic Hospital, Phoenix, AZ; b Department of Medicine, John A. Burns School of Medicine, University of Hawai'i, Honolulu, HI; c Department of Internal Medicine, Creighton University, Phoenix, AZ; d Department of Internal Medicine, Mayo Clinic, Phoenix, AZ

**Keywords:** atrial fibrillation, atrial flutter, cardiac arrhythmia, pulmonary arterial hypertension, supraventricular tachycardia

## Abstract

**Background:**

Cardiac arrhythmias can exacerbate symptoms and potentially lead to death in patients with pulmonary hypertension. However, there is limited evidence regarding the impact of cardiac arrhythmias in patients with pulmonary arterial hypertension (PAH).

**Research Question:**

What are the prevalence, incidence, and impact of arrhythmias in patients with PAH?

**Study Design and Methods:**

In a retrospective cohort study including 512 patients with PAH from 2001 to 2021 at 3 Mayo Clinic sites, demographic data at PAH diagnosis and clinical outcomes over a 10-year period were collected. The patients with PAH were categorized into 3 groups based on arrhythmic onset: (1) patients with arrhythmia before PAH diagnosis, (2) patients diagnosed with arrhythmia during PAH follow-up, and (3) patients without arrhythmia during PAH follow-up. Survival outcomes were analyzed using multivariable Cox proportional hazards regression, adjusted with the REVEAL 2.0 risk score.

**Results:**

Among the 512 patients with PAH (mean age, 56.1 years; 81.8% female), the prevalence of cardiac arrhythmias at PAH diagnosis was 10.5%, consisting of atrial fibrillation (7%), atrial flutter (2%), and supraventricular tachycardia (0.8%). The cumulative incidences of new-onset arrhythmias at 1, 5, and 10 years were 6%, 18%, and 29%, respectively. Patients with arrhythmia diagnosed before and after PAH diagnosis exhibited significantly higher all-cause mortality rates with adjusted hazard ratio of 2.06 (95% CI, 1.36-3.12) and 1.57 (95% CI, 1.17-2.20), respectively. Similarly, both arrhythmic groups demonstrated shorter median time to the first all-cause hospitalization (9.5 and 15.9 vs 21.2 months) and a higher number of all-cause hospitalizations (0.38 and 0.64 vs 0.10 times per year) compared with the nonarrhythmic group.

**Interpretation:**

Our results demonstrate that cardiac arrhythmias can develop in nearly one-third of patients with PAH within 10 years of PAH diagnosis and independently contribute to increased hospitalization frequency and mortality, in addition to the current REVEAL 2.0 risk score.

**Take-Home Points**

**Study Question:** What are the prevalence, incidence, and impacts of cardiac arrhythmias in patients with pulmonary arterial hypertension (PAH)?

**Results:** The prevalence of cardiac arrhythmias at PAH diagnosis is 10%. The cumulative incidences of new-onset arrhythmias at 1, 5, and 10 years after PAH diagnosis are 6%, 18%, and 29%, respectively. Patients with cardiac arrhythmia exhibited significantly higher all-cause mortality rates and a higher number of all-cause hospitalizations, whether present at PAH diagnosis or occurring later.

**Interpretation:** Cardiac arrhythmias in patients with PAH are common and significantly impact both morbidity and mortality. This study highlights the need for incorporating routine monitoring for arrhythmias into PAH patient care. Cardiac arrhythmias should be cooperated in prognostic risk stratification in patients with PAH.

Pulmonary arterial hypertension (PAH), known as group 1 pulmonary hypertension (PH), is a relatively rare condition characterized by remodeling distal pulmonary vasculature, mainly pulmonary arterioles, resulting in increased pulmonary vascular resistance (PVR) and elevated pulmonary artery pressure. Elevation of pulmonary pressure subsequently contributes to right ventricular (RV) remodeling and RV failure in response to increased RV afterload. PAH also leads to sympathetic overdrive in the autonomic nervous system and causes electrical remodeling of the right atrium and RV. These changes play an important role in arrhythmogenesis.^1^

Although the primary cause of death in the PAH population is RV failure as a consequence of progressively worsening RV hemodynamics,^2^, ^3^, ^4^ impacts of arrhythmias in this population are still under investigation. Atrial fibrillation (AF) and atrial flutter (AFL) are the most common types of arrhythmias in the PAH and chronic thromboembolic pulmonary hypertension (CTEPH) population, accounting for 10% to 33% of arrhythmias in these patients at some point in their lifetime.^5^, ^6^, ^7^, ^8^, ^9^, ^10^ Most of the previous studies have focused on AF and AFL. There are scattered data on all types of cardiac arrhythmia in the PAH population. The prevalence and incidence of arrhythmias in PAH are vague due to limited study numbers and diverse patient populations in each study.

Currently, the treatment of patients with PAH is directed by risk stratification tools, of which there are several aiming to evaluate survival rates based on a multiparametric approach. The recommended treatment goal for patients with PAH is to achieve and maintain a low-risk profile on optimized medical therapy.^11^, ^12^, ^13^ All of these risk stratification tools are composed of predictive variables including functional class status, 6-minute walk test (6MWT), and N-terminal pro B-type natriuretic peptide (NT-proBNP).^11^^,^^14^, ^15^, ^16^ The REVEAL 2.0 score is the most comprehensive risk stratification, which includes additional demographic characteristics and hemodynamic parameters. Although several studies have shown that cardiac arrhythmias are associated with clinical deterioration and increased mortality in patients with PH, none of the current risk stratification tools consider cardiac arrhythmias as a predictor.

In this study, we seek to provide a comprehensive analysis of all-type cardiac arrhythmias in terms of epidemiology and outcomes, especially on survival and all-cause hospitalization in the PAH population. Our objective is to investigate whether the presence of arrhythmias holds independent prognostic significance in PAH, beyond established risk stratification tools.

## Study Design and Methods

### Study Design and Population

This retrospective cohort study included 512 patients diagnosed with PAH at the 3 primary Mayo Clinic sites in Rochester (New York), Phoenix (Arizona), and Jacksonville (Florida) between 2001 and 2021. This study was approved by the institutional review board (study No. 19-002497).

This study attempted to include all adult patients with PAH encountered at the 3 Mayo Clinic sites according to the World Health Organization (WHO) pulmonary hypertension classification criteria for group 1 PH with idiopathic, connective tissue disease-associated, drug and toxin-induced, familial (heritable), pulmonary veno-occlusive disease, and HIV infection-associated PAH subtypes. Portal hypertension and congenital heart disease-associated PAH were not included due to distinct prognosis factors (eg, liver transplants, surgical correction of abnormal heart structures). The enrollment protocol started with identifying all group patients with PH using International Classification of Diseases, 9th Revision and International Classification of Diseases, 10th Revision codes diagnosed between January 2001 and December 2021. Right heart catheterization (RHC) data for these patients were then reviewed. We selected only the patients who met precapillary PAH at any time point during the study period, defined as a mean arterial pressure (mPAP) > 20 mm Hg, pulmonary arterial wedge pressure ≤ 15 mm Hg, and PVR > 2 Wood units per RHC hemodynamic definitions of PAH.^13^ Of patients with precapillary PH, we excluded patients who had left-sided heart disease, left ventricular ejection fraction < 40%, severe obstructive or restrictive lung disease, CTEPH, and group 5 PH. All patients’ clinical notes were reviewed manually to confirm the diagnosis of PAH. Patients with doubtful PH etiologies or multicomponents that contributed to PH were excluded from our studies. For connective tissue disease associated with PAH, we still included those patients who concomitantly developed mild and moderate interstitial lung diseases (ILDs) if degrees of pulmonary pressure were out-of-proportion to ILD severity determined by PH specialists. However, the patients with connective tissue disease associated with PAH who had severe ILD at the time of PAH diagnosis were excluded.

The demographic information was obtained by reviewing patients’ charts at the time of PAH diagnosis. Time zero for follow-up was defined from the date of RHC that met the PAH diagnosis. Echocardiographic data at the time of PAH diagnosis were obtained from the closest studies to patient time zero. This study followed patients for a maximum 10 years. The primary end point was all-cause death. We stopped following up on patients who underwent heart-lung transplants, and transplant patients were excluded from data analysis because heart-lung transplants had been done.

The patients were divided retrospectively into 3 groups based on arrhythmic status: (1) patients with PAH with arrhythmia(s) diagnosed at any time before PAH diagnosis, (2) patients with PAH with the first arrhythmia diagnosed during PAH follow-up, and (3) patients with PAH without arrhythmia. The diagnostic criteria for arrhythmias were the same across all groups, as subsequently described.

### Outcome Measures

Arrhythmias in our study were composed of AF, AFL, supraventricular tachycardia, ventricular tachycardia (VT), ventricular fibrillation (VF), sick sinus syndrome, second-degree atrioventricular block, and complete heart block. We subsequently classified patients with AF and AFL as paroxysmal and persistent AF/AFL. If a patient initially had paroxysmal AF/AFL but later met the definition of persistent AF/AFL during the study period, the patient was classified as persistent AF/AFL. The definition of all arrhythmias in the study strictly followed standardized definitions from the American College of Cardiology, American Heart Association, and Heart Rhythm Society.^17^, ^18^, ^19^, ^20^ A manual review of electrocardiograms and rhythm strips was done in every case to confirm the arrhythmic statuses. If an individual patient had multiple arrhythmias, each arrhythmia was reviewed and had the incidence calculated separately.

In addition to arrhythmia development, investigated outcomes during 10-year follow-up included the following: all-cause death, history of decompensated heart failure, time to first all-cause hospitalization, and average number of all-cause hospitalizations per year in each patient group. Decompensated heart failure in this study referred to worsening signs and symptoms of heart failure requiring additional interventions, including but not limited to medication change and cardioversion, documented by a cardiologist at any time during the study period. Hospitalized information was reviewed manually in electronic medical records. To obtain the most accurate count of hospitalizations, we counted the number of all-cause hospitalizations at the Mayo Clinic and other hospitals throughout the study period.

### Statistical Analysis

Continuous variables were reported as mean ± SD and median with interquartile range depending on distribution of data. Categorical variables were expressed as frequency with percentage. One-way analysis of variance, independent-samples Kruskal-Wallis test, and Pearson χ^2^ test were used followed by pairwise post hoc testing comparing between the 3 groups as appropriate. One minus the Kaplan-Meier survival function was selected to compute a cumulative incidence of arrhythmic development. In this model, patients with PAH with arrhythmia(s) diagnosed before PAH diagnosis were automatically censored, meaning that only patients with PAH who had not had any arrhythmias at the time of PAH diagnosis participated in this model. The time-to-event of each arrhythmic type was analyzed independently.

Crude 5- and 10-year survival times were obtained by the Kaplan-Meier survival function. The Cox proportional hazards regression model was used in survival analysis to compare all-cause deaths in each study group. The nonarrhythmic group served as a reference, and hazard ratios were adjusted by REVEAL 2.0 score at the time of PAH diagnosis, stratifying the patient into low-, moderate-, and high-risk groups. Subgroup analysis of association between all-cause death and paroxysmal and persistent AF/AFL used the Cox proportional hazards regression model. The nonarrhythmic group also served as a reference for AF/AFL characteristics survival analysis, and hazard ratios were adjusted by the onset of arrhythmia.

All statistical tests were performed by IBM SPSS Statistics version 28.0 (IBM Corp). *P* < .050 was considered statistically significant for all analyses.

## Results

### Population

We retrieved all RHC information from 5,828 adult patients who had an ICD-9 and ICD-10 diagnosis of PH between January 2001 and December 2021. There were 3,178 patients whose RHC met the criteria for diagnosis of precapillary PH. After the manual exclusion of PH WHO groups 2 through 5, portal hypertension, and congenital heart disease-associated PAH, a total of 512 patients with PAH including 264 with idiopathic PAH (51.6%), 212 with connective tissue disease-associated PAH (41.4%), 14 with drug- and toxin-induced PAH (2.7%), 14 with familial PAH (2.7%), 6 with pulmonary veno-occlusive disease (1.2%), and 2 with HIV infection-associated PAH (0.4%) were enrolled in our retrospective cohort study. In the cohort, 54 patients had at least 1 type of arrhythmia at the time of diagnosis, which was defined as group 1. Of the remaining 458 patients without arrhythmia at the time of diagnosis, 86 patients later developed arrhythmia(s) during the study period, defined as group 2. There were 372 patients who had no history of arrhythmia throughout the study period, and these patients were defined as group 3. Group 3 served as a control group during statistical analysis.

### Prevalence and Incidence of Arrhythmia

The prevalence of arrhythmia in the patients at the time of PAH diagnosis was 10.5%, and the prevalence of arrhythmia in follow-up of the entire cohort was 27.7% (Table 1). Most arrhythmias were AF, followed by AFL. Only a few patients had supraventricular tachycardia and VT at the time of PAH diagnosis, but a significant number of those arrhythmias developed during PAH follow-up, which accounted for 3.7% to 5.5% of the entire cohort. Approximately one-third of patients with arrhythmia experienced > 1 type of arrhythmia. Combinations of arrhythmic types are shown in e-Table 1.

**Table 1:** Crude Prevalence of Arrhythmias of the Entire Cohort

Prevalence of Arrhythmia	Total (n = 140)	Group 1: Arrhythmia(s) Diagnosed Before PAH Diagnosis (n = 54)	Group 2: Arrhythmia(s) Diagnosed During PAH Follow-Up (n = 86)
Total arrhythmia	140 (27.7)	54 (10.5)	86 (16.8)
Atrial fibrillation	99 (19.3)	37 (7.2)	62 (12.1)
Atrial flutter	42 (8.2)	13 (2.5)	29 (5.7)
Supraventricular tachycardia	28 (5.5)	4 (0.8)	24 (4.7)
Ventricular tachycardia	19 (3.7)	2 (0.4)	17 (3.3)
Ventricular fibrillation	3 (0.6)	0 (0.0)	3 (0.6)
Sick sinus syndrome	2 (0.4)	0 (0.0)	2 (0.4)
Second-degree AV block	8 (1.6)	1 (0.2)	8 (1.6)
Complete heart block	2 (0.4)	0 (0.0)	2 (0.4)
No. of arrhythmic types			
1	91 (65.0)	33 (61.1)	58 (67.4)
≥ 2	49 (35.0)	21 (38.9)	28 (32.6)

Values are No. (%). The percentages are arrhythmic proportions based on the entire cohort population. AV = atrioventricular; PAH = pulmonary arterial hypertension.

The cumulative incidence of arrhythmia development in the patients who did not have arrhythmia at the time of PAH diagnosis using the Kaplan-Meier approach was 6.6% over the first year after PAH diagnosis; then the incidence increased to 13.4%, 18.4%, and 29.2% over 3, 5, and 10 years, respectively. The cumulative incidences of each type of arrhythmia are displayed in Table 2.

**Table 2:** Cumulative Incidence of Arrhythmia Development Over Different Time Periods in Patients With PAH Who Had Never Had Any Arrhythmias at the Time of PAH Diagnosis

Cumulative Incidence^a^	1-y	3-y	5-y	10-y
Total arrhythmia	6.6	13.4	18.4	29.2
Atrial fibrillation	5.4	9.5	12.3	20.9
Atrial flutter	1.9	4.5	6.2	8.9
Supraventricular tachycardias	2.4	3.4	3.8	8.1
Ventricular tachycardia	1.0	2.1	3.5	5.9
Ventricular fibrillation	0.0	0.3	0.3	1.7
Sick sinus syndrome	0.2	0.2	0.2	0.9
Second-degree AV block	0.2	1.6	2.3	2.3
Complete heart block	0.0	0.5	0.5	0.5

Values are percentages. AV = atrioventricular; PAH = pulmonary arterial hypertension.

a The cumulative incidence calculations included only patients with PAH without a prior history of arrhythmias at the time of PAH diagnosis. Patients who had already been diagnosed with arrhythmias before their PAH diagnosis were automatically censored and excluded from the incidence calculations.

### Baseline Clinical Characteristics at the Time of PAH Diagnosis

Demographics and clinical characteristics of all 3 groups at the time of PAH diagnosis are demonstrated in Table 3. Most PAH etiologies were idiopathic and connective tissue disease-associated PAH. Although > 80% of the entire population was female, male proportions in the patients whose arrhythmia(s) were diagnosed either before or after PAH diagnosis were significantly higher than patients without arrhythmia. In comparison with patients without arrhythmia, those who had arrhythmia at any time tended to be older; have shorter 6MWT distance; have higher BNP; have higher REVEAL 2.0 scores; and have more comorbidities at the time of PAH diagnosis, including diabetes mellitus, hypertension, dyslipidemia, chronic kidney disease (estimated glomerular filtration rate < 60 mL/min/1.73 m^2^), and coronary artery disease. History of ischemic stroke was observed more often, up to 7.4% in group 1 patients who had a history of arrhythmia at the time of PAH diagnosis, compared with approximately 1% in patients without arrhythmia at the time of PAH diagnosis. No significant differences were noted between the 3 groups in baseline characteristics of race, PAH subtypes, number of PAH medications, mild and moderate lung diseases, liver cirrhosis, heart rate, oxygen saturation, WHO functional class, and diffusing capacity for carbon monoxide.

**Table 3:** Baseline Characteristics, Right Heart Catheterization, and Echocardiogram Parameters of Patients With PAH in Each Group at the Time of PAH Diagnosis

Parameters	Total (N = 512)	Group 1: Arrhythmia(s) Diagnosed Before PAH Diagnosis (n = 54)	Group 2: Arrhythmia(s) Diagnosed During PAH Follow-Up (n = 86)	Group 3: No Arrhythmia (n = 372)	*P* Value
Sex					.012
Female	419 (81.8)	40 (74.1)	63 (73.3)	316 (84.9)	
Male	93 (18.2)	14 (25.9)	23 (26.7)	56 (15.1)	
Age, y	56.1 [15.5]	65.8 [12.7]	57.7 [15.6]	54.3 [15.4]	< .001
Race					.157
White	450 (87.9)	45 (83.3)	83 (96.5)	322 (86.6)	
Asian	12 (2.3)	1 (1.9)	0 (0.0)	11 (3.0)	
Black	9 (1.8)	1 (1.9)	2 (2.3)	6 (1.6)	
Native American	6 (1.2)	1 (1.9)	1 (1.2)	4 (1.1)	
Others or unknown	35 (6.8)	6 (11.0)	0 (0.0)	29 (7.8)	
Ethnicity					.023
Hispanic or Latino	12 (2.3)	1 (1.8)	0 (0.0)	11 (3.0)	
Not Hispanic or Latino	368 (71.9)	42 (77.8)	72 (83.7)	254 (68.3)	
Unknown	132 (25.8)	11 (20.4)	14 (16.3)	107 (28.7)	
Subtypes of PAH					.786
Idiopathic	264 (51.6)	32 (59.3)	42 (48.8)	190 (51.1)	
Connective tissue disease-associated	212 (41.4)	22 (40.7)	39 (45.3)	151 (40.6)	
Drug- and toxin-induced	14 (2.7)	0 (0.0)	2 (2.3)	12 (3.2)	
Familial	14 (2.7)	0 (0.0)	2 (2.3)	12 (3.2)	
Pulmonary veno-occlusive disease	6 (1.2)	0 (0.0)	1 (1.2)	5 (1.3)	
HIV infection-associated	2 (0.4)	0 (0.0)	0 (0.0)	2 (0.5)	
PAH treatment					.124
No medication	4 (0.8)	0 (0.0)	1 (1.2)	3 (0.8)	
Monotherapy	143 (27.9)	19 (35.2)	16 (18.6)	108 (29.0)	
Double therapy	224 (43.8)	26 (48.1)	37 (43.0)	161 (43.3)	
Triple therapy	141 (27.5)	9 (16.7)	32 (37.2)	100 (26.9)	
Comorbidities					
Diabetes mellitus	56 (10.9)	14 (25.9)	15 (17.4)	27 (7.3)	< .001
Hypertension	138 (27.0)	25 (46.3)	26 (30.2)	87 (23.4)	.001
Dyslipidemia	96 (18.8)	19 (35.2)	17 (19.8)	60 (16.1)	.003
COPD	13 (2.5)	2 (3.7)	0 (0.0)	11 (3.0)	.247
OSA^a^	45 (8.8)	7 (13.0)	11 (12.8)	27 (7.3)	.137
Interstitial lung diseases^a^	94 (18.4)	9 (16.7)	18 (20.9)	67 (18.0)	.774
Coronary artery disease	19 (3.7)	3 (5.6)	7 (8.1)	9 (2.4)	.031
Ischemic stroke	10 (2.0)	4 (7.4)	1 (1.2)	5 (1.3)	.009
Liver cirrhosis	14 (2.7)	1 (1.9)	2 (2.3)	11 (3.0)	.869
BMI, kg/m^2^	29.2 [7.6]	31.3 [7.9]	27.7 [7.1]	29.2 [7.7]	.027
Systolic BP, mm Hg	119.7 [18.8]	124.5 [19.8]	116.5 [16.1]	119.7 [19.2]	.050
DiastolicBP, mm Hg	72.0 [11.6]	72.1 [12.5]	69.2 [10.4]	72.7 [11.7]	.044
Heart rate, beats per minute	82.6 [15.1]	78.5 [15.4]	83.9 [15.5]	82.9 [14.9]	.093
Oxygen saturation %	94.6 [4.1]	94.9 [3.3]	94.3 [4.3]	94.6 [4.2]	.722
WHO functional class					.063
I	49 (9.6)	5 (9.3)	5 (5.8)	39 (10.5)	
II	195 (38.1)	23 (42.6)	34 (39.5)	138 (37.1)	
III	229 (44.7)	17 (31.5)	39 (45.3)	173 (46.5)	
IV	39 (7.6)	9 (16.7)	8 (9.3)	22 (5.9)	
6-min walk test, m	328.1 [136.1]	262.6 [134.5]	312.9 [114.3]	339.9 [139.0]	.002
Diffusing capacity for carbon monoxide	57.9 [20.5]	56.7 [17.6]	58.8 [20.5]	57.8 [20.8]	.882
eGFR < 60 mL/min/1.73 m^2^	174 (34.0)	30 (55.6)	37 (43.0)	107 (28.8)	< .001
BNP, pg/mL, or NT-proBNP, pg/mL					.007
BNP < 50 or NT-proBNP < 50	78 (16.6)	1 (2.0)	13 (15.5)	64 (19.0)	
BNP 50-200 or NT-proBNP 50-300	136 (28.9)	12 (24.5)	17 (20.2)	107 (31.8)	
BNP 201-800 or NT-proBNP 301-1,100	140 (29.8)	19 (38.8)	31 (36.9)	90 (26.7)	
BNP ≥ 800 or NT-proBNP ≥ 1,100	116 (24.7)	17 (34.7)	23 (27.4)	76 (22.6)	
REVEAL 2.0 risk score					.001
Low (score ≤ 6)	469 (91.6)	45 (83.3)	73 (84.9)	351 (94.4)	
Intermediate (score 7-8)	36 (7.0)	9 (16.7)	10 (11.6)	17 (4.6)	
High (score ≥ 9)	7 (1.4)	0 (0)	3 (3.5)	4 (1.1)	
Right heart catheterization					
Right atrial pressure, mm Hg	10.3 [7.1]	13.8 [11.9]	10.3 [5.6]	9.7 [6.2]	< .001
Mean pulmonary arterial pressure, mm Hg	47.1 [13.1]	43.5 [11.8]	48.4 [12.8]	47.3 [13.3]	.081
Pulmonary arterial wedge pressure, mm Hg	10.1 [4.1]	12.3 [4.3]	9.9 [4.2]	9.9 [4.0]	< .001
Cardiac index, L/(min·m^2^)	2.4 [0.8]	2.3 [0.8]	2.4 [0.8]	2.4 [0.8]	.608
Cardiac output, L/min	4.4 [1.6]	4.4 [1.6]	4.5 [1.7]	4.4 [1.6]	.980
Pulmonary vascular resistance, WU	9.9 [6.4]	7.6 [4.2]	10.5 [6.7]	10.0 [6.6]	.021
Vasoreactivity testing					.531
Response	164 (39.9)	13 (33.3)	27 (37.0)	124 (41.5)	
Nonresponse	247 (60.1)	26 (66.7)	46 (63.0)	175 (58.5)	
Echocardiogram					
Right atrial area, cm^2^	21.7 [7.9]	29.1 [9.8]	23.4 [8.3]	20.4 [6.9]	< .001
Right ventricular area fraction change, %	31.3 [11.9]	32.8 [11.6]	27.9 [11.5]	32.2 [11.9]	.045
TAPSE, mm	19.5 [5.4]	19.5 [7.7]	19.5 [4.2]	19.5 [5.2]	.999
Left atrial area, cm^2^	18.2 [5.6]	23.6 [9.8]	17.6 [4.6]	17.6 [4.4]	< .001
LVEF, %	63.1 [8.3]	60.8 [10.0]	63.3 [9.6]	63.4 [7.6]	.095
Right ventricular systolic pressure, mm Hg	74.7 [24.5]	71.0 [22.0]	77.7 [22.7]	74.6 [25.3]	.283
LV diastolic dysfunction					.101
No diastolic dysfunction or grade 1	336 (89.1)	22 (81.5)	58 (84.1)	256 (91.1)	
Grade 2-4	41 (10.9)	5 (18.5)	11 (15.9)	25 (8.9)	
Tricuspid regurgitation					.137
No or trivial	131 (25.8)	8 (15.1)	21 (24.4)	102 (27.6)	
Mild	186 (36.6)	17 (32.1)	32 (37.2)	137 (37.1)	
Moderate	137 (27.0)	17 (32.1)	24 (27.9)	96 (26.0)	
Severe	54 (10.6)	11 (20.8)	9 (10.5)	34 (9.2)	
Pericardial effusion					.624
Presence	164 (32.9)	14 (26.9)	29 (34.1)	121 (33.4)	
No presence	335 (67.1)	38 (73.1)	56 (65.9)	241 (66.6)	
Timing of baseline echocardiogram					.170
Echocardiograms done before/at PAH diagnosis	333 (65.4)	38 (70.4)	49 (57.0)	246 (66.7)	
Echocardiograms done after PAH diagnosis	176 (34.6)	16 (29.6)	37 (43.0)	123 (33.3)	

Values are mean [SD], No. (%), or as otherwise indicated. BNP = B-type natriuretic peptide; eGFR = estimatedglomerular filtration rate; LV = left ventricular; LVEF = left ventricular ejection fraction; NT-proBNP = N-terminal pro B-type natriuretic peptide; PAH = pulmonary arterial hypertension; TAPSE = tricuspid annular plane systolic excursion; WHO = World Health Organization; WU = Wood units.

a Only mild and moderate severity of COPD, OSA, and interstitial lung diseases were included in the study. Degrees of mean arterial pressure in those patients must be out of proportion to lung diseases alone. Patients with advance stages of these comorbidities were excluded.

Regarding hemodynamic parameters, right atrial pressure and pulmonary arterial wedge pressure were higher in patients with arrhythmia at the time of PAH diagnosis. Interestingly, PVR in patients with arrhythmia at the time of PAH diagnosis was lower than PVR in patients without arrhythmias at the time of PAH diagnosis. Other RHC hemodynamic parameters and vasoreactivity responsiveness showed no difference between the 3 groups.

Baseline echocardiogram data at Mayo Clinic was obtained in 509 patients (99.4%). Of those, echocardiograms in 333 patients (65.4%) had been done before or at the time of PAH diagnosis by RHC, whereas echocardiograms in 176 patients (34.6%) had been done after the time of PAH diagnosis. The right and left atrial areas assessed by echocardiogram were larger in patients with arrhythmia. The RV area fraction change was significantly lower in the patients who developed arrhythmia during follow-up. There were no significant differences in other echocardiogram parameters (Table 3) between the 3 groups.

### Outcomes

The median follow-up time in this study was 4.21 years (interquartile range, 6.82), and the mortality of all groups was 39.5%. The arrhythmic groups showed higher mortalities, which were 53.7% in the patients with arrhythmia diagnosed before PAH diagnosis and 62.8% in the patients with arrhythmia diagnosed during PAH follow-up, compared with 32.0% in the patients without arrhythmia (Table 4). Within 10 years of the study period, 4.6% of the patients in the cohort underwent heart-lung transplantation. Crude 5-year survivals of groups 1, 2, and 3 were 60.6%, 55.5%, and 70.4%, respectively. Crude 10-year survivals of groups 1, 2, and 3 were 9.1%, 27.0%, and 56.1%, respectively.

**Table 4:** Descriptive Outcomes of Patients With PAH in Each Group During the Study Period

Outcomes	Total	Group 1: Arrhythmia(s) Diagnosed Before PAH Diagnosis (n = 54)	Group 2: Arrhythmia(s) Diagnosed During PAH Follow-Up (n = 86)	Group 3: No Arrhythmia (n = 372)	*P* Value
Follow-up time, median (IQR), y	4.21 (6.82)	2.7 (5.0)	4.7 (4.8)	4.3 (7.9)	.008
Death	202 (39.5)	29 (53.7)	54 (62.8)	119 (32.0)	< .001
Heart-lung transplant	23 (4.6)	1 (1.9)	12 (15.2)	10 (2.7)	< .001
History of decompensated heart failure	90 (17.6)	24 (44.4)	22 (25.6)	44 (11.8)	< .001
Time to the first all-cause hospitalization, median (IQR), mo	19.0 (45.8)	9.5 (22.3)	15.9 (42.6)	21.2 (48.8)	.004
Average number of all-cause hospitalizations per year, median (IQR)	0.20 (0.70)	0.38 (1.07)	0.64 (1.23)	0.10 (0.46)	< .001

Values are No. (%) or as otherwise indicated. IQR = interquartile range; PAH = pulmonary arterial hypertension.

**Figure 1:**
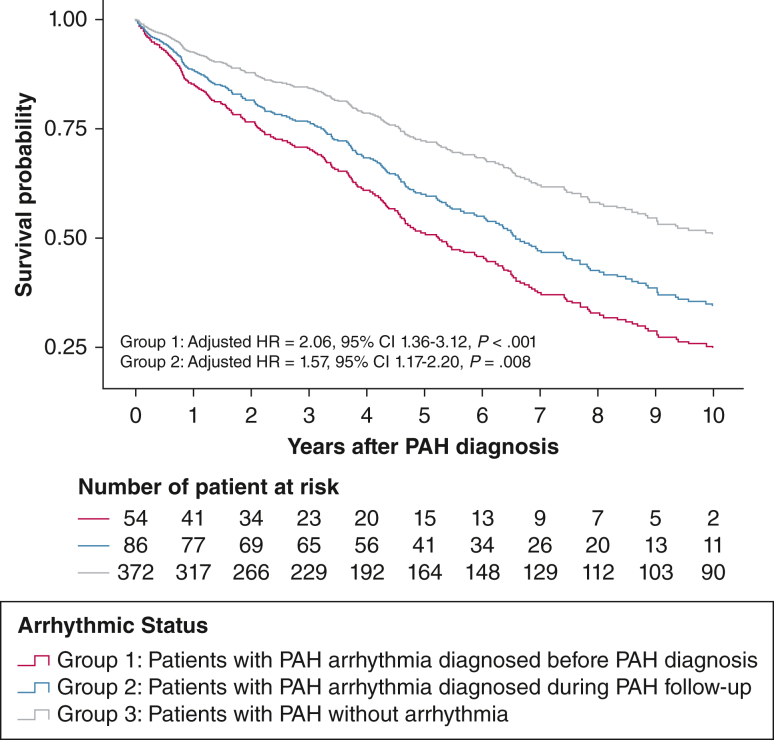
Cox proportional hazards curves showing the relation between 10-y survival of patients with PAH and arrhythmic status adjusted with REVEAL 2.0 score. HR = hazard ratio; PAH = pulmonary arterial hypertension.

Regarding the 10-year survival analysis, the estimated survival of patients with PAH substantially decreased in both arrhythmic groups (Fig 1). In univariate analysis by Cox proportional hazard regression, unadjusted hazard ratios were 2.34 (95% CI, 1.56-3.52; *P* < .001) and 1.93 (95% CI, 1.39-2.66; *P* < .001) in patients with arrhythmia diagnosed before PAH diagnosis and diagnosed during PAH follow-up, respectively. Because patients with arrhythmia had a lower REVEAL 2.0 score, the current prognostic risk score for PAH, the REVEAL 2.0 score, was chosen for hazard ratio adjustment. After the adjustment, arrhythmias were still significantly associated with worsened 10-year survival, as shown in Table 5. In a separate exploratory analysis, a subsequent hazard ratio adjustment was done with clinical covariates and hemodynamic parameters that were different in baseline characteristics, including age, male sex, and subgroups of PAH, mPAP, and PVR. The additional multivariate analysis still showed that arrhythmia was an independent risk factor for 10-year survival of the patients with PAH (e-Table 2).

**Table 5:** Associations Between 3 Arrhythmic Statuses and 10-Year Survival in Patients With PAH With Unadjusted Hazard Ratios and Adjusted Hazard Ratios With REVEAL 2.0 Score

Covariates	Univariate Analysis			Multivariate Analysis		
	HR	95% CI	*P* Value	HR	95% CI	*P* Value
REVEAL 2.0 risk score						
High (score ≥ 9)	5.98	(2.43–14.68)	< .001	5.61	(2.27-13.89)	< .001
Intermediate (score 7-8)	4.37	(2.94–6.51)	< .001	3.57	(2.36–5.39)	< .001
Low (score ≤ 6)	(1.00)		< .001	(1.00)		< .001
Arrhythmic status						
Arrhythmia diagnosed before PAH diagnosis	2.34	(1.56-3.52)	< .001	2.06	(1.36-3.12)	< .001
Arrhythmia diagnosed during PAH follow-up	1.93	(1.39-2.66)	< .001	<1.57	(1.13-2.20)	.008
No arrhythmia during PAH follow-up	(1.00)		< .001	(1.00)		< .001

HR = hazard ratio; PAH = pulmonary arterial hypertension.

In patients who experienced VT or VF, the mortality rate was notably high. Of the 22 patients diagnosed with VT or VF, 13 patients died during the follow-up period, with a median time to death after VT/VF diagnosis of 0.27 years (interquartile range, 3.2 years).

In addition, the prevalence of decompensated heart failure was higher in the arrhythmic groups. Compared with patients without arrhythmia, relative risks of having decompensated heart failure during PAH follow-up were 4.22 (95% CI, 2.63-6.76) and 2.04 (95% CI, 1.36-3.07) in patients with arrhythmia diagnosed before PAH diagnosis and diagnosed during PAH follow-up, respectively.

Arrhythmias had a negative impact on hospitalization as well. The patients with arrhythmia at the time of PAH diagnosis had the shortest median of time to first all-cause hospitalization, which was only 9.5 months compared with 21.2 months in patients without arrhythmia. Correspondingly, the average numbers of all-cause hospitalizations per year were higher in both arrhythmic groups.

### AF and AFL Population

**Figure 2:**
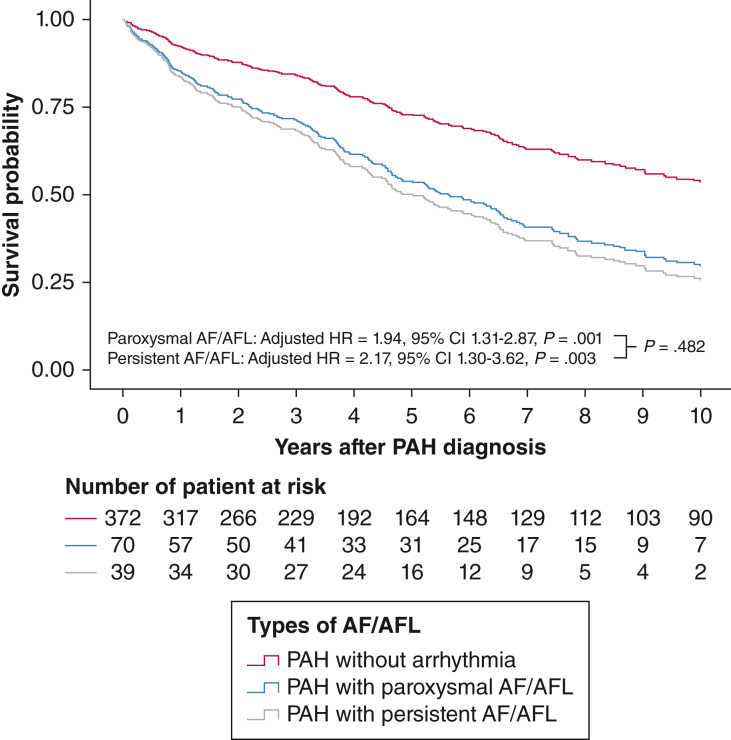
Cox proportional hazards curves showing the relation between 10-y survival of patients with PAH with paroxysmal and persistent AF/AFL compared with patients with PAH without arrhythmia adjusted with onset of the arrhythmias. AF = atrial fibrillation; AFL = atrial flutter; HR = hazard ratio; PAH = pulmonary arterial hypertension.

Due to the large number of patients with AF and AFL in the cohort, this study had the power to assess subgroup analysis of AF and AFL characteristics on PAH survival. There were 109 patients with PAH who were diagnosed with either AF, AFL, or both at any time during the study period. Of those, 39 patients had persistent AF or AFL, and 70 patients had paroxysmal AF or AFL. In a survival analysis using Cox proportional hazard regression adjusted with arrhythmic onset (Fig 2), both paroxysmal and persistent AF/AFL negatively impacted 10-year survival with adjusted hazard ratio of 1.94 (95% CI, 1.31-2.87; *P* = .001) for paroxysmal AF/AFL and adjusted hazard ratio of 2.17 (95% CI, 1.30-3.62; *P* = .003) for persistent AF/AFL. There was no statistically significant difference in survival between paroxysmal and persistent AF/AFL (log-rank *P* = .48).

## Discussion

This study with 512 patients with PAH demonstrated that cardiac arrhythmias are common and negatively impact outcomes.

### Prevalence and Incidence of Arrhythmias

Accurately determining the incidence and prevalence of PAH is challenging in the current literature due to the disease’s rarity, a small number of studies, and various included patient populations.^21^ Most of the reported incidence and prevalence from retrospective single-center analyses with a small population number result in decreased power to detect uncommon arrhythmias (eg, VT).

The prevalence of cardiac arrhythmias at the time of PAH diagnosis was found to be 10.5%. The cumulative incidence of new-onset arrhythmias at 1, 5, and 10 years was 6.6%, 18.4%, and 29.2%, respectively. These cumulative arrhythmic incidences include both atrial and ventricular arrhythmias, leading to slightly higher number of arrhythmic incidences than previous studies, which counted only atrial arrhythmias.^8^, ^9^, ^10^ Although a long-standing elevated pulmonary pressure results in RV dysfunction associated with RV fibrosis,^1^ the prevalence and incidence of VT have been poorly described in current literatures. Up to date, only 1 cross-sectional study stated a 2.3% prevalence of VT specifically in the PAH population.^22^ In our study, the crude prevalence of VT in the entire cohort is 3.7% and the cumulative incidence over 10 years is 5.9%. The discrepancy between the prevalence and the incidence suggests a significant death rate from VT soon after detection, which would limit the number of individuals who carry the VT diagnosis. These findings underscore the importance of considering arrhythmias as a significant clinical concern in the management of patients with PAH. Consideration should be given to routine monitoring and early intervention for arrhythmias in this patient population.

### Association of Baseline Clinical Characteristics and Arrhythmias

Regarding baseline characteristics, our study found that age, male sex, having diabetes, having hypertension, lower 6MWT, and BNP or NT-proBNP are associated with the presence of arrhythmia in PAH, similar to previous studies.^10^^,^^23^ It is well established that the incidence of AF increases with older age and differs between male and female individuals in the general population, with multiple studies showing a higher age-adjusted incidence of AF in male patients compared with female patients.^24^ Our study highlights that male sex and older age are associated with a higher prevalence of arrhythmias in patients with PAH, similar to the general population. In contrast to the 6MWT, which has been shown to correlate with arrhythmias in patients with PAH,^22^ the WHO functional class does not demonstrate a significant association with the presence of arrhythmias in the PAH population, as observed in this and previous studies.^7^, ^8^, ^9^^,^^23^ Our study also found that the arrhythmic groups had larger left and right atrial areas on echocardiogram than the nonarrhythmic group. In addition, multiple studies including this study consistently showed that the arrhythmic groups have higher right atrial pressure from RHC parameters compared with nonarrhythmic groups.^8^^,^^9^^,^^22^ The larger atrial areas may be evidence of atrial stretch and fibrosis from long-standing elevated atrium pressure, contributing to a substrate for arrhythmia.^1^ For other RHC parameters, mPAP is not different between arrhythmic and nonarrhythmic groups, and PVR tends to be lower in arrhythmic groups.^9^^,^^10^^,^^23^

### Clinical Outcomes and Mortality Associated With Cardiac Arrhythmias

Currently, arrhythmias are not included as a predictor in any of the survival risk prediction tools in patients with PAH.^11^, ^12^, ^13^, ^14^^,^^16^ However, cardiac arrhythmias have been recognized as contributing factors to clinical deterioration, involving elevated mortality and the potential for sudden death in patient with PAH.^1^^,^^10^^,^^25^

Mechanisms underlying clinical deterioration in cardiac arrhythmias include loss of adequate atrial contractions, atrioventricular dyssynchrony, and transient rapid heart rate, resulting in inability to maintain RV diastolic filling, especially with RV hypertrophy.^1^^,^^10^

The REVEAL 2.0 score, a well-established and widely used risk stratification tool in PAH management that has been validated in multiple studies, does not include arrhythmias as one of the survival risk predictors.^13^^,^^14^^,^^26^, ^27^, ^28^, ^29^, ^30^ However, an analysis of data from the Registry to Evaluate Early and Long-Term PAH Disease Management itself revealed a correlation between elevated heart rate and unfavorable outcomes.^31^

Patients with PAH are more vulnerable to hemodynamic change during atrial arrhythmias because AF and AFL disrupt atrioventricular synchronization. In the REVEAL registry, male individuals aged > 60 years are associated with higher mortality.^32^ Similarly, in this cohort, we found that male sex and older age are linked to a higher prevalence of arrhythmias and higher mortality (e-Table 2). Importantly, for adjusted hazard ratios of mortality, both with the REVEAL 2.0 score and with other covariates including age and male sex in our survival analysis, the presence of arrhythmias remained independently associated with increased mortality, highlighting that arrhythmias contribute additional risk beyond these demographic factors.

### Association With Heart Failure and Hospitalization

Hospitalizations in PAH are frequent, often attributed to cardiac conditions. In the Registry to Evaluate Early and Long-Term PAH Disease Management, > 50% of the patients with newly diagnosed PAH were admitted at least once within 3 years, with decompensated heart failure and arrhythmias accounting for almost 40% of these admissions.^33^ Similarly, among 207,095 hospitalizations of patients with PAH in the United States from 2001 to 2014, 48.5% were associated with cardiac issues. The primary causes of these stays were decompensated heart failure at 19.7%, followed by pulmonary heart disease at 9.4%, and dysrhythmias at 5.2%.

The arrhythmic groups had a substantially higher prevalence of decompensated heart failure during the follow-up period. Additionally, patients with arrhythmias, particularly those diagnosed at the time of PAH diagnosis, experienced a significantly shorter time to the first all-cause hospitalization and a higher frequency of hospitalizations compared with patients with nonarrhythmias. This emphasizes the impact of arrhythmias not only on mortality but also on the overall health care utilization and burden for patients with PAH.

### Subgroup Analysis of AF and AFL

Our study showed similar prevalence of AF and AFL comparted with previous studies, constituting 10% to 33% of the PAH and CTEPH population.^5^, ^6^, ^7^, ^8^, ^9^, ^10^^,^^34^ In our study, both paroxysmal and persistent AF/AFL significantly impacted 10-year survival. Wen et al^9^ showed that persistent supraventricular arrhythmias, including AF, AFL, and atrial tachycardia, increase mortality in PAH, similar to our study, whereas transient supraventricular arrhythmias are not associated with higher mortality. However, the paroxysmal AF/AFL population in our study is 3 times bigger than the previous study, which increases the statistical power to detect survival differences between the paroxysmal AF/AFL group and the nonarrhythmic group.

### Strengths and Limitations

This study had several strengths including a substantial sample size, comprehensive assessment of all cardiac arrhythmias, and extended 10-year follow-up period. However, there are still limitations in this study. Because most of the PAH population in this study had idiopathic PAH and connective tissue disease-associated PAH, the study’s generalizability may be limited to only both PAH subgroups. The study findings also cannot be applied in posttransplant patients with PAH because we terminated the patient follow-up after heart-lung transplantation. The prevalence and incidence of VT and VF may be understated due to survivorship bias caused by the deadly nature of the arrhythmias. The number of hospitalizations in this study also may be underreported. In a few cases, we could not obtain hospitalization history if patients were hospitalized outside Mayo Clinic network hospitals. Although this study is one of the biggest cohorts exploring arrhythmias in PAH, and this retrospective cohort provided strong associations between cardiac arrhythmia and increases in morbid and mortality in the PAH population, the nature of this study type could not explore whether the findings were directly causal or concomitant relationships. Another limitation of our study is the variability in the timing of baseline echocardiograms relative to RHC. In some cases, echocardiograms were obtained shortly after PAH-specific treatment was initiated, which may have influenced certain cardiac parameters. This reflects the practical challenges of observational studies, where data collection is often dependent on real-world clinical workflows. Standardizing the timing of echocardiograms in future studies could help address this limitation and provide more uniform insights.

### Implications for Clinical Practice and Future Research

The findings from our study have significant implications for clinical practice in the management of patients with PAH. Incorporating routine assessment of cardiac arrhythmias into the clinical evaluation and risk stratification of patients with PAH could help identify those at higher risk for adverse outcomes. Further research is needed to investigate the clinical outcomes of early detection and intervention for cardiac arrhythmia management.

## Interpretation

Our study contributes comprehensive insights into the epidemiology and clinical outcomes of cardiac arrhythmias in PAH. Our study reveals that 10.5% of patients with PAH have at least 1 type of arrhythmia at diagnosis, increasing to 29.2% after 10 years of follow-up. Arrhythmias, whether present initially or developing later, are independently associated with higher mortality, significantly worsening 10-year survival. The presence of AF or AFL is also associated with shorter time to hospitalization and more frequent hospitalizations. The findings underscore the need for a comprehensive approach to PAH management, integrating both hemodynamic and arrhythmic factors. Improved risk stratification tools and targeted interventions for cardiac arrhythmias in PAH are imperative for potentially enhancing patient outcomes.

## Funding/Support

The authors have reported to *CHEST Pulmonary* that no funding was received for this study.

## Financial/Nonfinancial Disclosures

None declared.

## Supplementary Data

e-Online Data

e-Online Data
