# Supplementary material for: Epidemiology and Clinical Outcomes of Cardiac Arrhythmias in Pulmonary Arterial Hypertension
Source: CHEST Pulm. 2024 Dec 25;3(1):100132. doi: 10.1016/j.chpulm.2024.100132 (PMC13418900; doi:10.1016/j.chpulm.2024.100132)
Supplement: e-Online Data [file mmc1.docx]

e-Table 1. The distribution of patients with combinations of arrhythmic types.

| **Combination of each type of arrhythmia** | **n, (%)**  **(N=140)** |
| --- | --- |
| Patients with at least 1 type of arrhythmias | 140 (100.0%) |
| AF | 55 (39.3%) |
| AF+AFL | 19 (13.6%) |
| AF+AFL+SVT | 3 (2.1%) |
| AF+AFL+SVT+VT | 1 (0.7%) |
| AF+AFL+SVT+2°AVB | 1 (0.7%) |
| AF+AFL+VT | 2 (1.4%) |
| AF+AFL+VF | 1 (0.7%) |
| AF+AFL+SSS | 1 (0.7%) |
| AF+AFL+2°AVB | 2 (1.4%) |
| AF+AFL+CHB | 1 (0.7%) |
| AF+SVT | 6 (4.3%) |
| AF+VT | 6 (4.3%) |
| AF+2°AVB | 1 (0.7%) |
| AFL | 9 (6.4%) |
| AFL+SVT | 1 (0.7%) |
| AFL+VT | 1 (0.7%) |
| SVT | 14 (10%) |
| SVT+VT | 2 (1.4%) |
| VT | 7 (5%) |
| VF | 2 (1.4%) |
| SSS | 1 (0.7%) |
| 2°AVB | 3 (2.1%) |
| 2°AVB+CHB | 1 (0.7%) |
| AF: Atrial fibrillation, AF: Atrial Flutter, SVT: Supraventricular tachycardia, VT: Ventricular tachycardia, VF: Ventricular fibrillation, SSS: Sick sinus syndrome, 2°AVB: Second-degree AV block, CHB: Complete heart block | |
