# Supplementary material for: Epidemiology and Clinical Outcomes of Cardiac Arrhythmias in Pulmonary Arterial Hypertension
Source: CHEST Pulm. 2024 Dec 25;3(1):100132. doi: 10.1016/j.chpulm.2024.100132 (PMC13418900; doi:10.1016/j.chpulm.2024.100132)
Supplement: e-Online Data [file mmc2.docx]

e-Table 2. Associations between 3 arrhythmic statuses and 10-year survival in PAH patients with unadjusted hazard ratios and adjusted hazard ratios with age, male sex, PAH subgroups, mean pulmonary arterial pressure, and pulmonary vascular resistance.

|  | **Univariate analysis** | | |  | **Multivariate analysis** | | |
| --- | --- | --- | --- | --- | --- | --- | --- |
| **Covariate** | **HR** | **95% CI** | ***P* value** |  | **HR** | **95% CI** | ***P* value** |
| Age (per 10-year increased) | 1.38 | (1.25–1.53) | <0.001 |  | 1.31 | (1.17–1.47) | <0.001 |
| Male | 1.59 | (1.14–2.22) | 0.007 |  | 1.69 | (1.18–2.42) | 0.004 |
| Subgroup of PAH IPAH PAH-CTD Others | (1.00) 1.84 0.57 | (1.39–2.45) (0.26–1.22) | <0.001 <0.001 0.146 |  | (1.00) 2.33 0.78 | (1.69–3.21) (0.36-1.70) | <0.001 <0.001 0.532 |
| mPAP | 1.01 | (0.99–1.02) | 0.086 |  | 1.01 | (0.99–1.02) | 0.223 |
| PVR | 1.04 | (1.02–1.06) | <0.001 |  | 1.05 | (1.02–1.08) | <0.001 |
| Arrhythmic status Arrhythmia diagnosed before  PAH diagnosis Arrhythmia diagnosed during  PAH follow-up No arrhythmia during PAH follow-up | 2.34  1.93  (1.00) | (1.56–3.52)  (1.39–2.66) | <0.001  <0.001  <0.001 |  | 1.89  1.42  (1.00) | (1.20–2.98)  (1.02–1.98) | 0.006  0.039  0.009 |
